# Measuring affect and complex working memory in natural and urban environments

**DOI:** 10.3389/fpsyg.2023.1039334

**Published:** 2023-03-06

**Authors:** Emily E. Scott, Kaedyn W. Crabtree, Amy S. McDonnell, Sara B. LoTemplio, Glen D. McNay, David L. Strayer

**Affiliations:** ^1^Department of Psychological Science, Vermont State University, Johnson, VT, United States; ^2^Department of Psychology, The University of Utah, Salt Lake City, UT, United States; ^3^Human Dimensions of Natural Resources, Colorado State University, Fort Collins, CO, United States

**Keywords:** attention restoration theory, working memory, affect (emotion), environment, cognitive performance, restoration

## Abstract

**Introduction:**

Research suggests that spending time in natural environments is associated with cognitive and affective benefits, while increased use of technology and time spent in urban environments are associated with depletion of cognitive resources and an increasing prevalence of mental illness. Attention Restoration Theory suggests that exposure to natural environments can restore depleted attentional resources and thereby improve cognitive functioning and mood. Specifically, recent meta-analyses have revealed that the most improved cognitive abilities after nature exposure include selective attention, working memory, and cognitive flexibility.

**Methods:**

While existing studies examined these cognitive abilities, few have examined the Operation Span (OSPAN), a complex measure of working memory capacity. Therefore, the current study (*N* = 100) compared performance on the OSPAN and self-reported mood using the Positive and Negative Affect Schedule before and after a 30-min walk in a natural or urban environment.

**Results:**

Results from the study showed that both groups exhibited an increase in positive affect and a decrease in negative affect, suggesting that going outside for a walk can boost mood regardless of environment type. Inconsistent with past work, there were no significant changes in OSPAN scores before and after the walk for either environment type.

**Discussion:**

Future studies should analyze how the length of time spent in the environment, certain characteristics of the environment, and individual differences in connectedness to nature may impact attention restoration to gain insight on nature’s ability to improve our affect and cognition.

## 1. Introduction

It is projected that by the year 2050, upwards of 70% of the world’s population will reside in urban centers (United Nations, Department of Economic and Social Affairs, Population Division, 2019). There are many benefits to living in a city; however, prior research suggests that urban living can have negative impacts on physical health, mental health, and even on our neurobiology (for review, see Lambert et al., 2015). Living or spending prolonged time in cities can induce stress, fatigue, and mental illness (Groenewegen et al., 2006; Peen et al., 2010; Lederbogen et al., 2011; Min et al., 2017) due to taxing environments, stimulus overload, and repeated exposure to technology (Afifi et al., 2018; Jakobsson et al., 2019). These stressors can lead to depletion of cognitive resources, such as attention (Kaplan, 1995). Attention plays a crucial role in problem solving, conflict resolution, suppressing distracting stimuli, short-term memory (Jonides et al., 2008), and successful cognitive and emotional function (Berman et al., 2008). To succeed in these everyday tasks, it is beneficial to restore attention after it has been depleted from stressful or cognitively demanding tasks at work, school, and from technology.

One hypothesized way to restore attention is outlined by Attention Restoration Theory (ART) which states that attention can be restored by spending time in nature (Kaplan, 1995). Previous studies have shown that exposure to natural environments can reduce self-reported stress (Beil and Hanes, 2013; Roe et al., 2013; Scott et al., 2020), increase positive affect (Beute and De Kort, 2014; Bratman et al., 2015; Lee et al., 2015), induce relaxation (Anderson et al., 2017) and increase cognitive performance across a range of processes such as creativity (Atchley et al., 2012), sustained attention (Hartig et al., 2003), and working memory (Tennessen and Cimprich, 1995; Kuo and Sullivan, 2001; Ottosson and Grahn, 2005; Berman et al., 2008, 2012).

Recent meta-analyses (Ohly et al., 2016; Stevenson et al., 2018) suggest that improvements in cognition apply most strongly to tasks that engage working memory, such as the forwards or backwards digit-span task. These tasks require holding items (a series of digits in forwards or backwards order) in memory while drawing on attentional resources to maintain these stimuli while ignoring distracting information (Unsworth and Robison, 2017). The Operation Span task (OSPAN), which involves holding a string of letters in memory while completing simple math problems, is a reliable measure of WMC (Unsworth et al., 2005). While ample studies have explored the effect nature has on working memory performance on forward digit-span (Tennessen and Cimprich, 1995; Ottosson and Grahn, 2005) and backward digit-span tasks (Berman et al., 2008, 2012; Kuo and Sullivan, 2001), only one study, to our knowledge, has used the Operation Span task (OSPAN) to measure working memory performance associated with exposure to nature (Bratman et al., 2015).

Importantly, research suggests that while “complex” span tasks such as OSPAN, and “simple” span tasks share variance, they are also likely measuring separable components of working memory abilities (e.g., Colom et al., 2006; Kane et al., 2007; Unsworth and Engle, 2007; Arnell et al., 2010). The OSPAN draws strongly upon attentional inhibitory control (Engle and Kane, 2004; Kane et al., 2007), as individuals are introduced to distracting math problems between working memory sets (Unsworth et al., 2005). This distracting component of the task is absent in most versions of digit span tasks. Therefore, it is unclear whether the OSPAN task would demonstrate the same improvements in performance due to nature as the digit span task reliably does (Stevenson et al., 2018). Therefore, the aim of this study was to examine whether previous findings on digit span performance after nature exposure extend to more complex working memory performance as assessed by the OSPAN, and to replicate findings by Bratman et al. (2015). Bratman et al. (2015) analyzed OSPAN scores in 45 participants before and after either a 50-min nature walk or a 50-min urban walk and found an average increase in OSPAN scores by about 10 points (or around 27%) from pre-post nature walk but not pre-post urban walk (Bratman et al., 2015).

Furthermore, Bratman et al. (2015) found improvements in mood after nature exposure. Specifically, they found that negative affect decreased and positive affect increased after the nature walk compared to the urban walk. This finding is consistent with accumulating evidence that spending time in nature reliably improves mood (for review see McMahan and Estes, 2015). These benefits seem to extend to exposures as short as 5 min (Neill et al., 2019), and do not appear to be influenced by season of the year (Brooks et al., 2017). Therefore, we included the PANAS questionnaire (Watson et al., 1988), a commonly used measure of affect, to further replicate these findings, hypothesizing that we would also observe an improvement in mood after the nature walk but not the urban walk.

Our study seeks to replicate and expand these findings with a larger sample size (*N* = 100) and new environmental conditions. We employed a mixed subjects experimental design commonly observed in the nature literature (e.g., Berman et al., 2008; Bratman et al., 2015) with pre-post measurements (within subjects) and urban-nature walk (between subjects) components. We reduced the walk to 30 min to examine whether we could replicate findings of cognitive benefits in nature with a shorter walk duration, as this length of time may be more feasible and accessible for the average person. Additionally, many studies in the field contain sample sizes that may not be large enough to detect reliable or even small effects (Stevenson et al., 2018). For example, previous work has suggested that the effect size for working memory improvements in nature is *g* = 0.162 (95% CI = 0.053–0.270)—a small effect size (Stevenson et al., 2018). According to power analyses conducted in PANGEA (Westfall, 2015), we should need anywhere from 80 to 500 participants depending on the variance to detect an effect this small. We aim to replicate prior working memory and nature studies with a larger and more statistically powered sample size.

Lastly, while some previous studies assume that participants enter the study with depleted attentional resources (Bratman et al., 2015; Gidlow et al., 2016), our study ensures this by inducing the depletion of cognitive resources before participants leave on their walk with a proofreading task commonly used in the ego-depletion literature (Muraven and Baumeister, 2000). This follows best practices outlined by Stevenson et al. (2018) in their recent review and meta-analysis. Therefore, the current study provides insight to whether natural environments can be used as a tool to restore attentional capacity and boost mood after cognitive resources have been depleted. We hypothesized that participants assigned to a nature walk would show an improvement in OSPAN score from before their walk to after their walk, as well as perform significantly better after their walk compared to participants assigned to an urban walk.

## 2. Materials and methods

### 2.1. Participants

Participants were recruited through an undergraduate course at the University of Utah (*N =* 100). Of the participants, 75 identified as female, 24 as male, and one as non-binary ranging between 18 and 46 years old (*M =* 23.36, *SD =* 5.01). Participants completed one 1.5-h session and received course credit for participation in the study. Participants were randomly assigned to either a nature condition (*N =* 50) or an urban condition (*N =* 50) during the study. An *a priori* power analysis was conducted through GPower (Faul et al., 2007) which determined that 50 participants were needed for each condition to detect between-within interactions, based on 80% power and a medium effect size of *d* = 0.50 (Cohen, 1998). There were no significant differences in age or gender between the two conditions (*p*’s > 0.57). All participants included had normal or corrected-to-normal vision, had no neurological disorders, and had to be physically able to complete the walk.

### 2.2. Measures

#### 2.2.1. Attention depletion task

To accurately measure attention restoration, it is common in the literature to first deplete attentional resources using a proofreading task (Baumeister et al., 1998). The proofreading task that was utilized in this study involved crossing out the letter “e” in a long text while following a specific set of complex rules (see Baumeister et al., 1998). This task has been used to deplete self-regulatory resources, such as attention, prior to a task assessing attentional abilities (Muraven and Baumeister, 2000). Ensuring depletion of these resources is a common practice in the literature to fully examine attention restoration and to ensure that all participants left on their walks in a comparably cognitively depleted state (Beute and De Kort, 2014).

#### 2.2.2. Positive and negative affect schedule

The PANAS, a self-report survey measuring state-based changes in mood, requires participants to rate 20 different emotional states based on a series of words (e.g., inspired, attentive, afraid, determined) on a scale from 1 (not at all) to 5 (extremely; Watson et al., 1988; Watson and Clark, 1994). These words were categorized and summed into positive and negative affect categories, with higher scores reflecting greater affect. The PANAS is one of the most utilized scales for assessing mood in the nature and cognition literature.

#### 2.2.3. Operation span task

The standard administration of the automated OSPAN task involves completing sets of simple math problems while simultaneously remembering and recalling a series of randomly generated letters (Unsworth et al., 2005). In the task, a string of letters appears one at a time (i.e., T) after a simple math problem appears (i.e., 9 × 3–2 = 25) on a computer screen, programmed in E-prime 2.0. Participants choose ‘True’ or ‘False’ to answer the math problem before another letter appears. After a series of math problems and letters, participants are then asked to recall the letters in the order they appeared in. Participants are encouraged to respond as quickly and accurately as possible but must maintain 85% math accuracy, and participants completed practice trials before beginning the task (Unsworth et al., 2005). While the OSPAN is most often used to assess trait working memory capacity for use in individual differences studies, the OSPAN has also been shown to be sensitive to experimental manipulations (Curci et al., 2013; Bratman et al., 2015; Luo et al., 2017).

### 2.3. Procedure

Data were collected in the Sugarhouse neighborhood within Salt Lake City, Utah comprising of a small natural area and an abundance of shopping centers in the larger urban neighborhood. All data were collected between the hours of 9 am–4 pm and took place in October. Data were collected in varying weather conditions and ranged between 30 degrees Fahrenheit and 60 degrees Fahrenheit with an average of 48 degrees. Participants were set in a weatherproof enclosed pod with chairs and access to space heaters. Upon arrival, participants read and signed the consent document before completing a participant information and demographics form. Participants then received instructions for and completed the depletion task for 10 min. Once complete, participants filled out the PANAS assessing their mood in the current state, then moved on to complete the OSPAN task on a laptop, which took approximately 20 min to complete. The depletion task, PANAS, and OSPAN were all completed in an outdoor location that was located equidistant (about 20 yards) from the start point of both walking routes.

**Figure 1:**
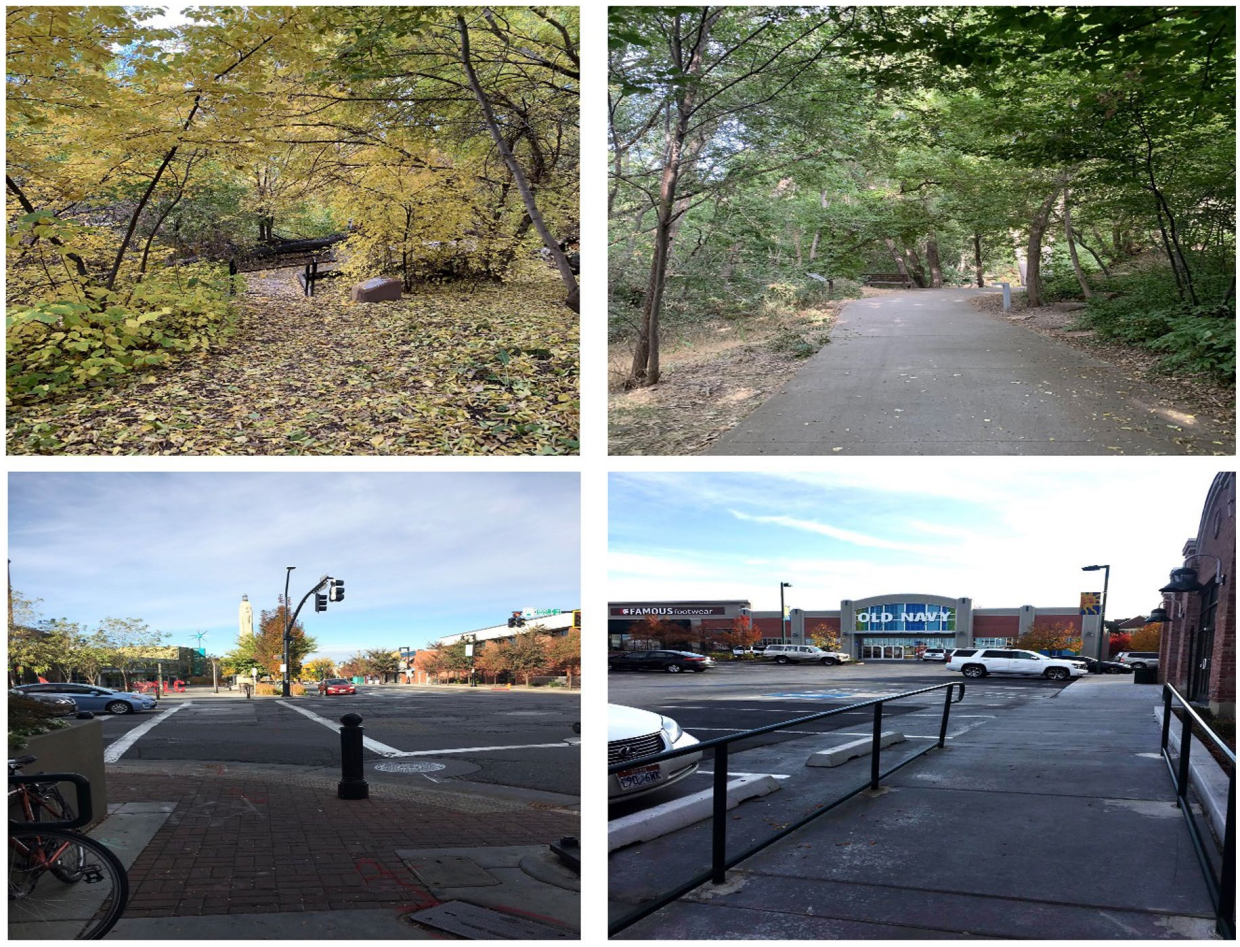
Images of the nature walk (top) and urban walk (bottom).

Participants were then randomly assigned to go on either a walk through the natural area, or a walk through the urban city environment within the Sugarhouse neighborhood located within Salt Lake City. Both walks consisted of a predetermined route which created a loop, which participants were asked to complete twice. Both the nature and urban walk took approximately 30 min to complete. Both routes were of equivalent distance and took place on flat, paved sidewalks (urban condition; see Figure 1) or flat, dirt pathways (nature condition; see Figure 1). Both routes were evenly populated as they were within close proximity.

**Figure 2:**
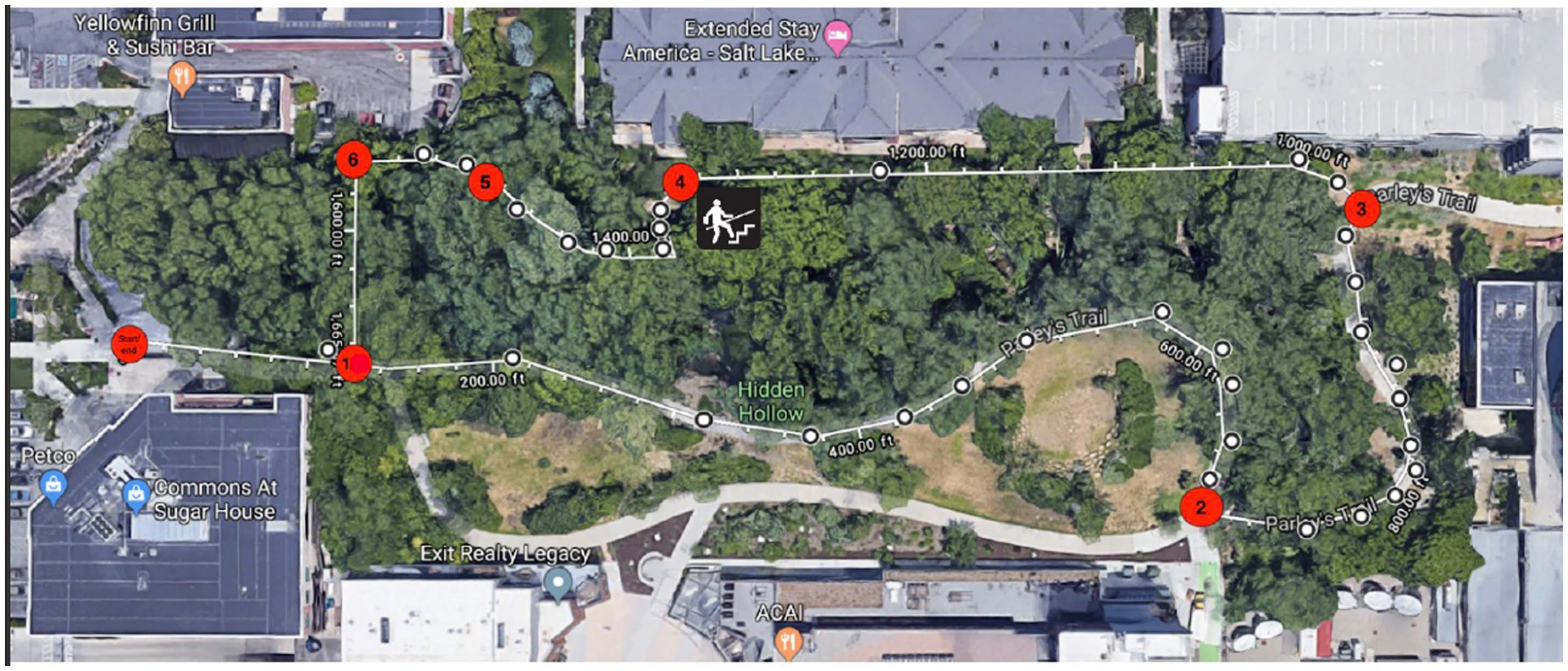
Participant map for the nature condition. Source: Map data @2023 Google

**Figure 3:**
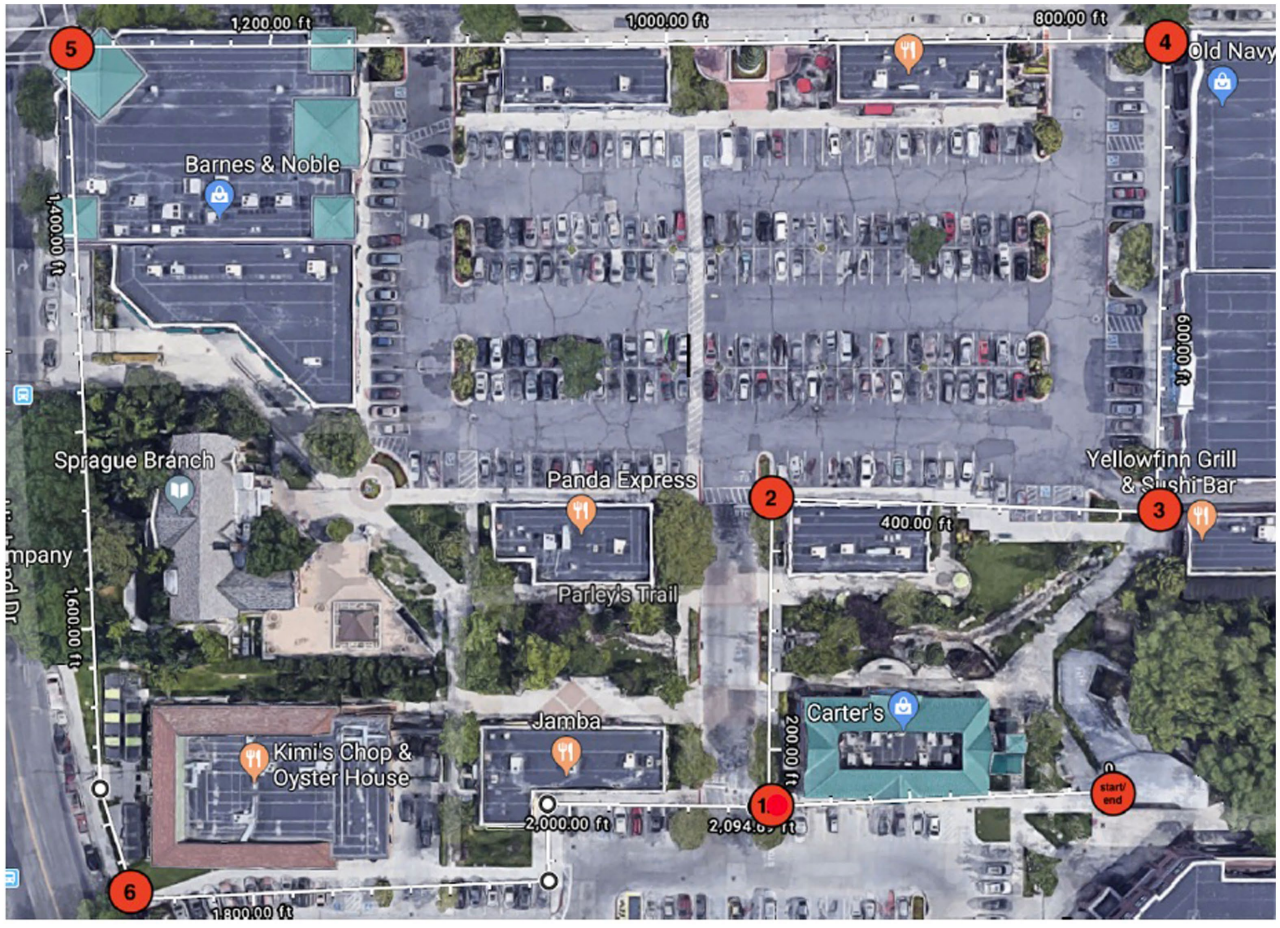
Participant map for the urban condition. Source: Map data @2023 Google

In both the nature and urban condition, participants were each given a map showing the route of the walk they were to complete. On each map (see Figures 2, 3) there were 6 ‘checkpoints’ where participants could refer to correlating written directions telling them where to go if they came to a fork in the road, or a turn to avoid getting lost. At each of the 6 “checkpoints” participants were asked to take a picture on their phone of something interesting at that checkpoint, and then pictures were checked by a researcher at the end of their walk. This manipulation check was to ensure all participants did not deviate from the predetermined route, replicating procedures from Bratman et al. (2015). All participants were encouraged to be present in their environments by turning their phone on airplane mode to discourage distractions. Research assistants instructed participants to walk at their normal pace and take breaks when needed. Upon completion of the walk, participants completed the PANAS and the OSPAN task for a second time, and then were debriefed by the research team and given course credit.

#### 2.3.1. Environmental conditions

Participants assigned to the nature condition completed their walk through a small nature walk area within the Sugarhouse neighborhood. The nature area included an abundance of trees, a river, various species of plants, and several bridges (see top left and right pictures in Figure 1). Participants assigned to the urban condition completed their walk through a small downtown portion of the Sugarhouse area. This walk included walking alongside busy streets lined with stores, restaurants, and parking lots (see bottom left and right pictures in Figure 1). Participants in this condition were instructed to stay on the sidewalk and utilize crosswalks when necessary.

### 2.4. Statistical analyses and hypotheses

We examined two main *a priori* hypotheses in this study. First, we predicted an interactive effect of testing session and environmental condition on OSPAN score, such that participants would improve in their OSPAN performance from pre-post walk, but only in the nature condition and not the urban condition. We predicted a similar interaction with the outcome variable of mood, such that positive affect would increase and negative affect would decrease from pre-post walk in both conditions, due to mood-boosting effects of exercise (e.g., Lane and Lovejoy, 2001; Lane et al., 2002), but that this increase would be more pronounced in the nature condition. To test these hypotheses, we ran repeated measures ANOVAs in R’s version 4.0.3, with session and environment as two predictive, interactive terms. Post-hoc comparisons were conducted using Turkey multiple comparison of means.

## 3. Results

### 3.1. PANAS

**Figure 4:**
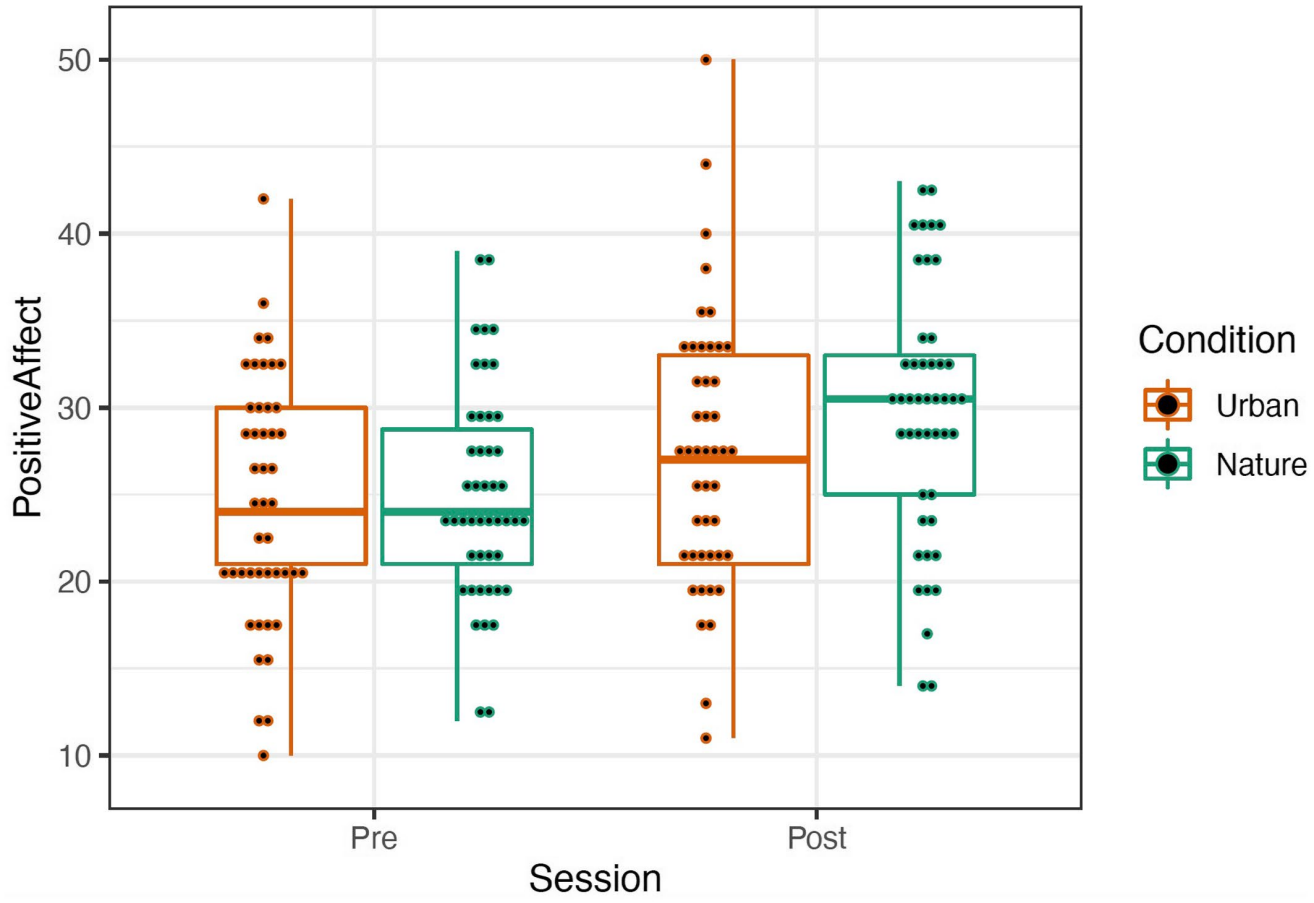
Positive (left) Affect represented by a boxplot. The smaller dots represent individual data.

**Figure 5:**
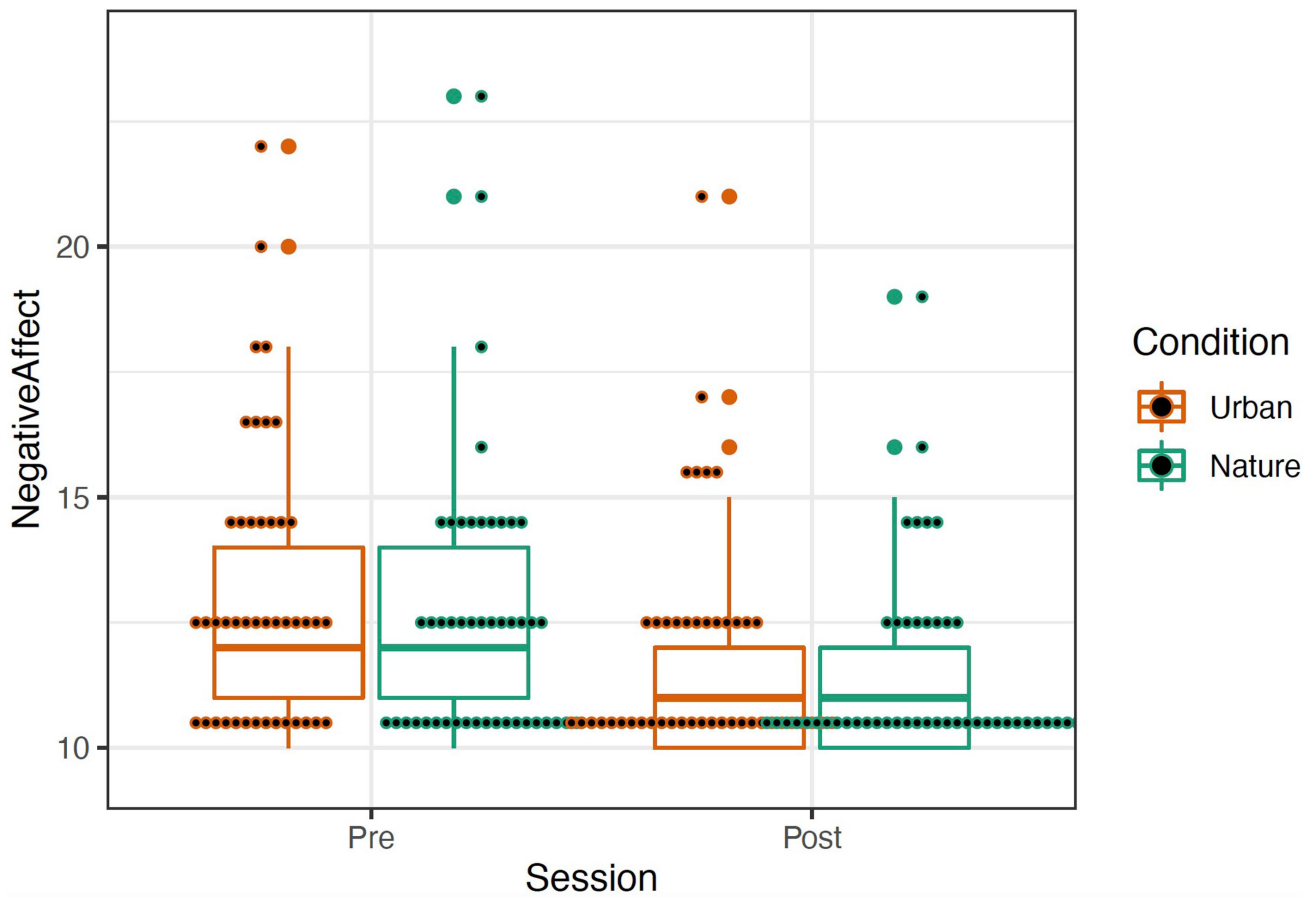
Negative (right) Affect represented by a boxplot. The larger dots indicate outliers, and the smaller dots represent individual data. Removal of outliers did not significantly affect results; thus, they were left in the dataset.

The PANAS was scored by summing items describing positive and negative affect (Watson and Clark, 1999; see Table 1). A repeated-measures mixed ANOVA was conducted to examine the effects of condition (Nature and Urban), time (Pre-walk and Post-walk), and the condition by time interaction on the affect categories. Levene’s tests confirmed equal group variances (*p*’s > 0.39), and independent samples *t*-tests confirmed that there were no significant differences between conditions in average scores at the Pre-walk baseline for positive (*t*(89) = −0.24, *p* = 0.81) and negative (*t*(89) = 1.56, *p* = 0.12) affect. ANOVA results revealed that there was no significant main effect of condition for either positive or negative affect, *p*’s > 0.292. There was a significant effect of time in both the positive affect, *F*(1, 95) = 40.413, *p* < 0.001, and the negative affect categories, *F*(1, 95) = 31.891, *p* < 0.001, such that participants in both groups increased in positive affect and decreased in negative affect over time (see Figures 4, 5). There were no significant effects for a time by condition interaction for either positive or negative affect, *p*’s > 0.348. Data were analyzed in R Version 4.0.3 (Table 1).

**Table 1:** Mean PANAS scores and standard deviations for pre-walk and post-walk conditions.

PANAS	Nature		Urban	
	Pre	Post	Pre	Post
Positive affect	25.03 (6.08)	30.0 (7.18)	24.88 (6.67)	27.65 (7.56)
Negative affect	12.61 (2.74)	11.29 (1.87)	13.27 (3.01)	11.82 (2.53)

**Table 2:** Mean OSPAN scores and standard deviations for pre-walk and post-walk conditions.

OSPAN	Nature		Urban	
	Pre	Post	Pre	Post
Partial	59.09(12.47)	61.27(12.16)	59.4(12.2)	59.22(13.19)
Absolute	44.41 (17.3)	47.36(17.73)	44.09 (17.59)	47.58(16.26)

### 3.2. OSPAN

With respect to data loss, eleven participants were removed due to technical issues that resulted in file loss or incomplete data (9) and failure to complete the second OSPAN task (2), resulting in a sample of 89 participants for the OSPAN analysis. Individual files were merged into a single combined E-prime file and data were analyzed in R Version 4.0.3.

**Figure 6:**
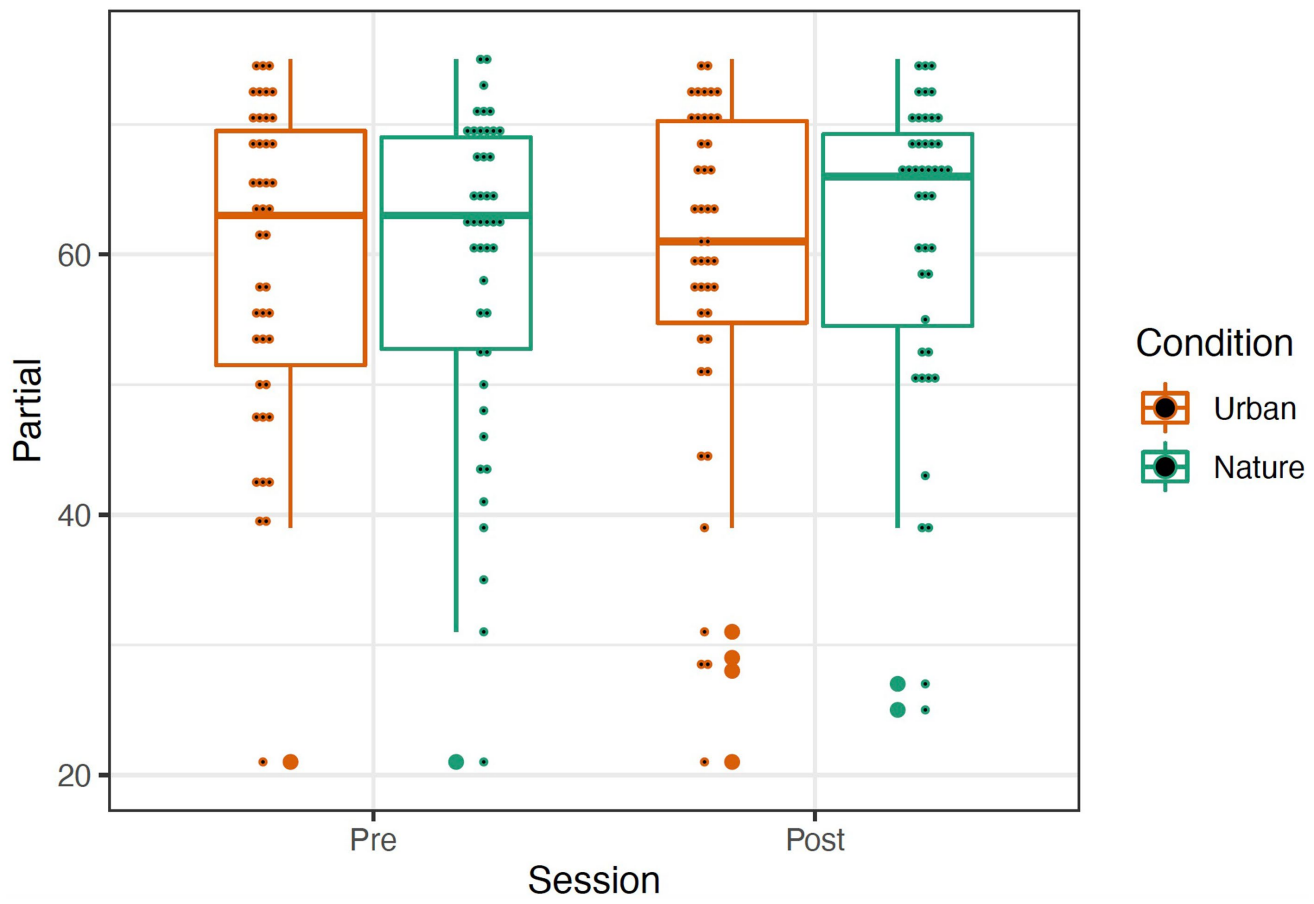
Partial (left) OSPAN scores represented by a boxplot. The larger dots indicate outliers, and the smaller dots represent individual data. Removal of outliers did not significantly affect results; thus, they were left in the dataset.

**Figure 7:**
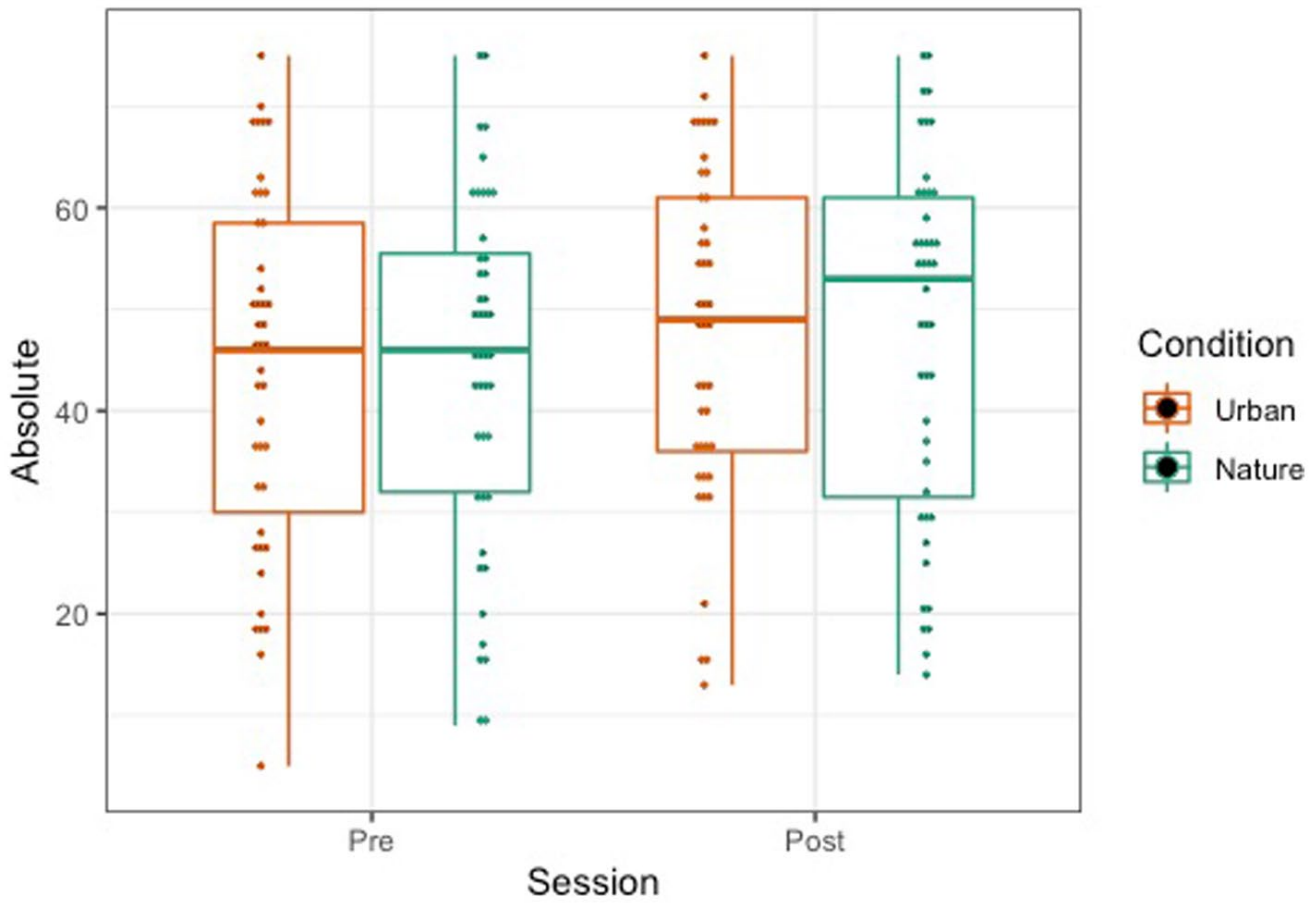
Absolute (right) OSPAN scores represented by a boxplot. The smaller dots represent individual data.

The OSPAN score was calculated by the sum of all perfectly recalled sets (absolute score or “all or nothing” method), meaning all letters and their serial order were recalled correctly within a set. We also examined the total number of letters recalled in the correct position (partial score or “total number correct” method). A repeated-measures mixed ANOVA was conducted to examine the effects of condition (Nature and Urban), time (Pre-walk and Post-walk) and the condition by time interaction on the performance of the partial and absolute components of the OSPAN score. Levene’s tests confirmed equal group variances (*p*’s = 0.41), and independent samples *t*-tests confirmed that there were no significant differences between conditions in average scores at the Pre-walk baseline for partial (*t*(89) = −0.39, *p* = 0.69) and absolute (*t*(89) = −0.48, *p* = 0.63) OSPAN scores. There were no significant main effects of time, condition, or a time by condition interaction on either partial or absolute OSPAN performance (see Figures 6, 7; all *p*’s > 0.189).

## 4. Discussion

The current study measured differences in self-reported affect and OSPAN performance before and after going on a walk in an urban or natural environment. In line with previous work (for review see Ohly et al., 2016; Frumkin et al., 2017; Stevenson et al., 2018), we hypothesized that the nature group would experience an increase in positive affect and decrease in negative affect and show improvements on the OSPAN task compared to the urban group.

We found that there was an overall increase in positive affect and a decrease in negative affect in both groups after going on their outdoor walk. These findings, consistent with other studies (Gidlow et al., 2016), imply that simply going on a walk outside can boost your mood regardless of the environment type. Similarly, Berman et al. (2012) also found a decrease in negative affect after a 50-min walk, regardless of environment type. We did not have an indoor walking condition or a neutral, non-walking condition, so we cannot determine if these findings are simply due to being outside, taking a break from cognitively stimulating tasks, the exercise benefits of the walk, or some interaction of these components. We also found no difference in working memory performance as measured by the OSPAN, indicating that neither nature nor urban environments influence complex working memory span performance. There are many possible explanations to why we found no difference in OSPAN or PANAS scores between the nature and urban groups. Below, we discuss these findings in the context of prior work.

### 4.1. OSPAN

One interpretation of the results could be that the OSPAN as a working memory capacity measure is insensitive to nature exposure compared to other working memory measures. Research found that there is a weak correlation between digit-span tasks and OSPAN tasks, indicating that findings between the two tasks are not directly comparable to one another (Kane et al., 2007; though see Unsworth et al., 2007). Similarly, others suggest that digit span tasks are a measure of pure working memory capacity, whereas complex span tasks involve other higher order processes such as attentional inhibition (Diamond, 2013). As prior research has shown improvements in digit span performance after nature exposure (Tennessen and Cimprich, 1995; Kuo and Sullivan, 2001; Ottosson and Grahn, 2005; Berman et al., 2008, 2012), perhaps some mechanism the OSPAN captures (i.e., recall) is not as reliably affected by exposure to nature as other cognitive tasks. Indeed, this hypothesis is also supported by a recent meta-analysis that found weaker nature effects on tasks designed to measure cognitive inhibition compared to simple span tasks (Stevenson et al., 2018). Future work should more explicitly examine the extent to which inhibitory components of working memory tasks are influenced by exposure to nature. For example, researchers could directly compare how different working memory tasks respond to natural environments: the OSPAN task, the digit span task, and a digit span task that includes a more potent inhibitory manipulation.

We aimed to replicate Bratman et al. (2015) findings of an increase in OSPAN performance after nature exposure. One intention of our study was to decrease the walk duration from 50-min to 30-min to work towards a minimal possible “dose” of nature that might be sufficient to improve complex working memory capacity. The reasoning behind this intention was to increase accessibility for the average individual that may experience barriers (i.e., time, physical, or geographic barriers) to accessing nature. This intention was validated by prior research that has reported improvements in mood and attention with a 30-min walk or less (Martens et al., 2011; Gidlow et al., 2016; Shrestha et al., 2021). However, in the original study (Bratman et al., 2015), participants walked for 50-min. Therefore, one possible explanation for our null results is that this dosage of nature exposure was insufficient to influence complex working memory capacity. We did see slight but non-significant changes in average OSPAN scores at post-testing for both nature (absolute: 6.6% increase, partial: 3.7% increase) and urban groups (absolute: 7.9% increase, partial: −0.03% decrease), which could be due to practice effects. However, given that Bratman and colleagues found a 27% increase in OSPAN performance, it is possible that longer durations of nature exposure may be needed to improve complex working memory capacity.

Finally, it is also possible that our photo-taking manipulation check, though in line with our attempt at replication (Bratman et al., 2015), may have had depleting effects on attentional performance. Because we did not want to induce anxiety by removing phones in a novel or unfamiliar environment altogether, we instructed participants to keep their phones on airplane mode as the use of a phone can hijack attention and cause distraction (Thornton et al., 2014; Stothart et al., 2015). While it is important to include some method of verifying strict adherence to the route chosen for experimental control, future studies might include other methods of accomplishing this goal. For example, prior nature and cognition research in real-world settings include GPS watches to track location (i.e., Berman et al., 2008, 2012). We suggest that if the use of phones was depleting for attentional performance, it would also show depleting effects on affect based on research suggesting a link between affect or stress and cognitive performance (Thayer and Lane, 2000; Scott et al., 2021). However, our results showed improved mood for all conditions.

### 4.2. PANAS

Interestingly, our study showed improvements in negative and positive affect scores for both nature and urban groups. An improvement in mood for both environmental conditions has been demonstrated in the nature literature previously (Gidlow et al., 2016). There may be several reasons why we also saw no differences in mood between the nature and urban conditions. For example, it is possible that the depletion task lowered positive mood and increased negative mood compared to a participants’ baseline mood and that after the walk, we simply observed a return to baseline in mood. However, we note that the depletion task is meant to emulate everyday demands on attention, such as studying for an exam or a draining day at work. In this case, if an individual is feeling depleted, our results suggest that walking outdoors may improve mood.

We speculate that enhanced mood in nature is more likely to occur in more dramatic natural environments, such as a remote wilderness area. The natural area chosen in our study has some built components (i.e., a wooden bridge over a creek). However, these environments may be more representative of life in an urban city and therefore likely have greater ecological validity. Additionally, these environments are similar to previous research that we aimed to replicate (i.e., Berman et al., 2008, 2012). That said, research that explicitly examines how working memory is affected by mixed urban and natural elements could be an interesting future direction, considering most of our world is composed of mixed environments. There is some pre-existing research on this topic (e.g., McAllister et al., 2017; Yu et al., 2018; Brancato et al., 2022), but limited research that examines cognitive outcomes such as working memory specifically. Importantly, the current study suggests that taking a short walk outside can improve mood.

Finally, we propose that improvements in affect in nature may be a necessary precursor to improvements in higher-order cognitive processes such as working memory. For example, prior work suggests that there is a causal relationship between state anxiety and working memory performance, such that anxiety or worry can interrupt performance on working memory tasks (for review see Moran, 2016). Therefore, is possible that we did not see improvements in the OSPAN task in nature compared to urban environments *because* there were no differential improvements in mood in nature. This would be consistent with recent theory suggesting that improvements in attention and stress or affective variables in nature are linked *via* the vagus nerve (Thayer and Lane, 2000; Scott et al., 2021), which connects the heart and the brain. Future work is necessary to further understand the relationship between mood improvements and cognitive improvements in nature.

### 4.3. Experimental design

Many previous real-world studies involving nature walks have a comparable experimental design as our study but had smaller sample sizes, which could potentially lead to overestimations of effect sizes (Button et al., 2013) and subsequently affect replicability (Open Science Collaboration, 2015). This study supports growing non-replications of behavioral findings in the nature and attention literature (i.e., see Johnson et al., 2021; Neilson et al., 2021). However, behavioral measures are not the sole indicator of attention restoration. Recent research suggests that nature exposure may also result in attentional and affective changes at the neural level (Grassini et al., 2019, 2022; Hopman et al., 2020; LoTemplio et al., 2020; Sudimac et al., 2022), which may or may not be revealed behaviorally. For example, methods from neuroscience such as electroencephalography (EEG) and functional magnetic resonance imaging (fMRI) can capture changes in real-time brain activity during a cognitive task that do not always correspond to changes in behavioral responses.

Future studies should analyze how duration, and level of nature exposure (e.g., imagery versus real-world environments) affect different measures of cognitive restoration with complimentary measures of performance (e.g., subjective, behavioral, and physiological). Although our results are not consistent with prior work, there is strong evidence that duration of exposure and season of exposure have minimal effects on mood improvements in nature (Brooks et al., 2017; Neill et al., 2019). However, it is unclear whether this is the case for cognitive outcomes such as working memory performance. Therefore, future work might explicitly compare how variations in “dosage” of nature affect working memory performance. To this end, researchers may also consider using within-subject designs that have participants experience both urban and nature conditions. This within-subject, multi-session design could add to the validity of the experiment by helping to control for within-subject variability. Studies could also have multiple routes per condition to ensure robustness of the condition effect, rather than the route itself.

Lastly, it is worth noting the possibility that there were untested individual differences that moderated the relationship between environmental exposure and both working memory and affect. For example, as all the participants lived in the city or surrounding area in which the study was conducted, it is possible that some were familiar with the environments and therefore habituated to them. It is also possible that there could be other unknown individual differences that impact these results. For example, an individual’s self-reported connectedness to nature (Mayer and Frantz, 2004) is known to moderate perceived restoration in nature (e.g., Berto et al., 2018). It is currently unknown how individual differences such as connectedness to nature might moderate the relationship between nature and various cognitive variables such as working memory. Therefore, this represents an interesting topic for future research.

## 5. Conclusion

As our world becomes increasingly urban, it is important to understand the psychological benefits of natural spaces in urban areas. This study suggests that short durations of urban nature exposure may not be enough to improve complex span tasks such as the OSPAN, but that they may reliably improve mood. This result is somewhat surprising, as it conflicts with a prior study demonstrating improvements OSPAN performance following nature (e.g., Bratman et al., 2015), and strong prior work demonstrating both mood improvements in nature (McMahan and Estes, 2015) and improvements in other working memory capacity measures such as digit span (Stevenson et al., 2018). We note that it is possible that this result emerged due to differences in the OSPAN task compared to digit span tasks. Therefore, future work should explicitly compare how nature affects both complex and simple measures of working memory capacity within the same sample. Additionally, future work is necessary to address both the relationship between affect and working memory in nature, and the “dosage” of nature necessary to improve working memory performance.

## Data availability statement

The raw data supporting the conclusions of this article will be made available by the authors, without undue reservation.

## Ethics statement

The studies involving human participants were reviewed and approved by Institutional Review Board at The University of Utah. The patients/participants provided their written informed consent to participate in this study.

## Author contributions

ES and KC were involved in all phases of the research, including study design, data collection and management, analyses, and manuscript writing. AM, SL, and GM contributed to data collection and manuscript writing. DS contributed to study design. All authors contributed to manuscript editing and approved the submitted version.

## Funding

Open access fees are covered by DS’s professional development funds at the University of Utah.

## Conflict of interest

The authors declare that the research was conducted in the absence of any commercial or financial relationships that could be construed as a potential conflict of interest.

## Publisher’s note

All claims expressed in this article are solely those of the authors and do not necessarily represent those of their affiliated organizations, or those of the publisher, the editors and the reviewers. Any product that may be evaluated in this article, or claim that may be made by its manufacturer, is not guaranteed or endorsed by the publisher.
